# A Preclinical and Phase Ib Study of Palbociclib plus Nab-Paclitaxel in Patients with Metastatic Adenocarcinoma of the Pancreas

**DOI:** 10.1158/2767-9764.CRC-22-0072

**Published:** 2022-11-02

**Authors:** Manuel Hidalgo, Rocio Garcia-Carbonero, Kian-Huat Lim, Wells A. Messersmith, Ignacio Garrido-Laguna, Erkut Borazanci, Andrew M. Lowy, Laura Medina Rodriguez, Daniel Laheru, Beatriz Salvador-Barbero, Marcos Malumbres, David J. Shields, Joseph E. Grossman, Xin Huang, Meggan Tammaro, Jean-François Martini, Yanke Yu, Kenneth Kern, Teresa Macarulla

**Affiliations:** 1 Hematology and Medical Oncology, Weill Cornell Medicine, New York, New York.; 2 Oncology Department, Hospital Universitario 12 de Octubre, Imas12, UCM, CNIO, CIBERONC, Madrid, Spain.; 3 Department of Medicine, Division of Oncology, Washington University School of Medicine, St. Louis, Missouri.; 4 School of Medicine, University of Colorado Cancer Center, Aurora, Colorado.; 5 Department of Oncology, Huntsman Cancer Institute, University of Utah, Salt Lake City, Utah.; 6 HonorHealth/TGen, Scottsdale, Arizona.; 7 Department of Surgery, UC San Diego Moores Cancer Center, San Diego, California.; 8 Hospital Universitario de Fuenlabrada, Fuenlabrada, Madrid, Spain.; 9 Department of Oncology, The Sidney Kimmel Comprehensive Cancer Center at Johns Hopkins, Baltimore, Maryland.; 10 School of Biosciences, European Cancer Stem Cell Research Institute, Cardiff University, Cardiff, United Kingdom.; 11 Cell Division and Cancer Group, Spanish National Cancer Research Centre (CNIO), Madrid, Spain.; 12 Centers for Therapeutic Innovation, Pfizer Inc, New York, New York.; 13 Department of Medicine, Beth Israel Deaconess Medical Center, Harvard Medical School, Boston, Massachusetts.; 14 Pfizer Oncology, San Diego, California.; 15 Gastrointestinal Cancer Unit, Vall d´Hebrón University Hospital and Vall d´Hebrón Institute of Oncology (VHIO), IOB Quirón, Barcelona, Spain.

## Abstract

**Purpose:**

To assess the preclinical efficacy, clinical safety and efficacy, and MTD of palbociclib plus nab-paclitaxel in patients with advanced pancreatic ductal adenocarcinoma (PDAC).

**Experimental Design:**

Preclinical activity was tested in patient-derived xenograft (PDX) models of PDAC. In the open-label, phase I clinical study, the dose-escalation cohort received oral palbociclib initially at 75 mg/day (range, 50‒125 mg/day; modified 3+3 design; 3/1 schedule); intravenous nab-paclitaxel was administered weekly for 3 weeks/28-day cycle at 100‒125 mg/m^2^. The modified dose–regimen cohorts received palbociclib 75 mg/day (3/1 schedule or continuously) plus nab-paclitaxel (biweekly 125 or 100 mg/m^2^, respectively). The prespecified efficacy threshold was 12-month survival probability of ≥65% at the MTD.

**Results:**

Palbociclib plus nab-paclitaxel was more effective than gemcitabine plus nab-paclitaxel in three of four PDX models tested; the combination was not inferior to paclitaxel plus gemcitabine. In the clinical trial, 76 patients (80% received prior treatment for advanced disease) were enrolled. Four dose-limiting toxicities were observed [mucositis (*n* = 1), neutropenia (*n* = 2), febrile neutropenia (*n* = 1)]. The MTD was palbociclib 100 mg for 21 of every 28 days and nab-paclitaxel 125 mg/m^2^ weekly for 3 weeks in a 28-day cycle. Among all patients, the most common all-causality any-grade adverse events were neutropenia (76.3%), asthenia/fatigue (52.6%), nausea (42.1%), and anemia (40.8%). At the MTD (*n* = 27), the 12-month survival probability was 50% (95% confidence interval, 29.9–67.2).

**Conclusions:**

This study showed the tolerability and antitumor activity of palbociclib plus nab-paclitaxel treatment in patients with PDAC; however, the prespecified efficacy threshold was not met.

**Trial Registration:**

Pfizer Inc (NCT02501902)

**Significance:**

In this article, the combination of palbociclib, a CDK4/6 inhibitor, and nab-paclitaxel in advanced pancreatic cancer evaluates an important drug combination using translational science. In addition, the work presented combines preclinical and clinical data along with pharmacokinetic and pharmacodynamic assessments to find alternative treatments for this patient population.

## Introduction

Pancreatic ductal adenocarcinoma (PDAC) is a very aggressive malignancy associated with substantial morbidity and mortality (1). The median overall survival (OS) for patients with metastatic pancreatic cancer is approximately 12 months. The current standard of care for advanced disease is systemic chemotherapy (2). The combination of leucovorin, fluorouracil, irinotecan, and oxaliplatin (FOLFIRINOX) is used as a first-line treatment option for patients with good performance status (1). Although the FOLFIRINOX regimen significantly improved median OS versus gemcitabine (11.1 vs. 6.8 months; *P* < 0.001), the safety profile was substantially worse than that of gemcitabine (3), and many patients are not healthy enough to receive FOLFIRINOX (4). Gemcitabine plus nab-paclitaxel (GA) is another first-line treatment option for patients with metastatic pancreatic cancer that demonstrated clinical benefit versus gemcitabine alone (median OS, 8.5 vs. 6.7 months; *P* < 0.001) and a manageable safety profile (1). In December 2019, the PARP inhibitor olaparib was approved as maintenance treatment for patients with BRCA-mutated PDAC (5, 6).

An urgent need exists for new effective treatments for patients with PDAC. However, despite encouraging preclinical and early clinical data, agents such as pegilodecakin (7) and PEGPH20 (8) were not efficacious in randomized clinical trials. Furthermore, the role of immunotherapy in treating this disease remains to be determined; strategies tested thus far have not been effective (9).

One of the predominant genetic alterations in PDAC is inactivation of the *CDKN2A* locus (∼80% of cases) encoding the cyclin-dependent kinase 4/6 (CDK4/6) inhibitors p16^INK4A^ and p14^ARF^, which leads to the aberrant activation of the CDK4/6 complex and cell proliferation (10–12). CDK4/6 are serine/threonine kinases that modulate cell-cycle entry by phosphorylation of the retinoblastoma protein (RB1), thereby inhibiting its transcriptional repression function (13). These kinases are frequently activated in human cancer either by overexpression of their activating subunits, D-type cyclins, or inactivation of CDK4/6 inhibitors of the INK4 protein family (14). Because of their important function in cell-cycle entry and G_1_ progression, CDK4/6 inhibitors are considered therapeutic targets in several tumor types (13). Palbociclib, a CDK4/6 inhibitor, is approved in combination with endocrine therapy to treat hormone receptor–positive/HER2-negative advanced breast cancer (15, 16). Several preclinical studies have shown that CDK4/6 inhibitors exhibit an antiproliferative effect in PDAC cell lines and patient-derived xenografts (PDX; refs. 17–19). Palbociclib induces cell-cycle arrest and apoptosis in PDAC cell lines that retain RB1 expression (17). The combination of this agent with gemcitabine decreased the incidence of liver metastases and extended survival in PDX models (17).

On the basis of these data, we conducted a series of preclinical studies to test the efficacy and optimal combination of palbociclib in PDX models. The results of these studies indicated that palbociclib plus nab-paclitaxel was more effective than GA and not inferior to the triple drug combination. Subsequently, a phase Ib clinical trial was conducted to evaluate the safety, pharmacokinetics, pharmacodynamics, preliminary efficacy, and MTD of palbociclib plus nab-paclitaxel in patients with advanced PDAC.

## Materials and Methods

### Preclinical Studies

All mouse procedures carried out were previously approved by the Centro Nacional de Investigaciones Oncológicas Institutional Animal Care and Use Committee, as well as the Bioethics Committee of the Instituto de Salud Carlos III and Comunidad de Madrid. The methods for the preclinical studies are detailed in Supplementary Materials and Methods.

### Clinical Trial Design

This was an open-label, multicenter, dose escalation, safety, pharmacokinetics, pharmacodynamics, and preliminary efficacy study of palbociclib plus nab-paclitaxel in patients with advanced PDAC. The primary objective of this trial was to assess the safety and tolerability of palbociclib in combination with nab-paclitaxel in patients with advanced PDAC to estimate the MTD and select the recommended phase II dose. In the dose-escalation phase, patients received oral palbociclib starting at 75 mg/day and ranging from 50 to 125 mg/day (based on dose escalation and de-escalation), for 21 of every 28 days (3/1 schedule) plus nab-paclitaxel (Supplementary Fig. S1). Intravenous nab-paclitaxel was administered at 100–125 mg on days 1, 8, and 15 every 28 days. In cycle 1, nab-paclitaxel was administered on day ‒2 to evaluate the pharmacokinetic of nab-paclitaxel administered alone versus with palbociclib. The criteria for dose escalation were based on a modified toxicity probability method (20), using a statistical probability algorithm calculated with all patients treated at the same dose level. In the two modified dose–regimen cohorts, patients received palbociclib 75 mg/day (3/1 schedule or continuously) plus nab-paclitaxel (biweekly 125 or 100 mg/m^2^, respectively). After the MTD of palbociclib plus nab-paclitaxel was estimated from the dose-escalation phase, patients were enrolled into an MTD expansion cohort. The study was conducted in accordance with legal and regulatory requirements and the Declaration of Helsinki. Prior to participant enrollment, the study was approved by the Institutional Review Board/Ethics Committee at each site at which it was conducted. Patients provided written informed consent before any study procedures were performed.

### Patients

Eligible patients had histologically or cytologically confirmed PDAC with radiographically confirmed metastatic disease; were ages ≥18 years (ages ≤75 years for the dose-escalation cohort); had a Karnofsky performance status of ≥70; and had adequate bone marrow, renal, and liver functions. With the exception of nab-paclitaxel, prior therapies for treating disease were permitted in the dose escalation and modified dose–regimen cohorts. Archived tumor tissue (or de novo biopsy specimen if no archived tumor tissue was available) for biomarker analysis was required (Supplementary Materials and Methods). Exclusion criteria included known central nervous system metastases, carcinomatous meningitis, or leptomeningeal disease; a QTc >480 ms; a history of long or short QT syndrome; Brugada syndrome; QTc prolongation; Torsade de pointes; and uncontrolled electrolyte disorders.

### Outcomes

The primary study outcome was first-cycle dose-limiting toxicities. Other outcomes included safety, pharmacokinetics, pharmacodynamics, tissue/blood biomarkers, objective tumor response, progression-free survival (PFS), and OS assessments. Adverse events (AE) were graded by the NCI Common Terminology Criteria for Adverse Events version 4.03. The pharmacokinetic and pharmacodynamic activities of palbociclib and nab-paclitaxel were evaluated on the basis of plasma samples collected and analyzed on day 1 of cycle 1 during the lead-in phase when nab-paclitaxel was administerd alone and on day 13 of cycle 1 when nab-paclitaxel was coadministered with palbociclib.

To measure cancer antigen 19-9 (Ca 19-9), blood samples were collected and analyzed at a local laboratory. Changes in phospho-Rb (pRb) and Ki67 in paired skin biopsy specimens over the course of two cycles of palbociclib treatment were analyzed using IHC staining (Supplementary Materials and Methods).

Objective tumor response was assessed using the RECIST version 1.1. Objective response rate (ORR) was defined as the percentage of evaluable patients with confirmed complete response (CR) or partial response (PR). Clinical benefit rate was defined as the percentage of patients with a best overall response of CR or PR at any time or stable disease for ≥16 weeks from the first day of treatment.

On the basis of the 48% 12-month survival rate observed in the phase I/II trial (21), the prespecified efficacy target was set at a 12-month survival probability of ≥65% at the MTD. The MTD was defined as the dose associated with <33% of 9 patients experiencing a dose-limiting toxicity.

Exploratory endpoints included analysis in plasma cell-free DNA (cfDNA) of *CDKN2A*, *RAS*, and *TP53* mutations to evaluate possible associations with resistance or sensitivity to treatment (Supplementary Materials and Methods).

### Statistical Analyses

Using the Kaplan–Meier method, PFS and OS were summarized; the median event time and two-sided 95% confidence interval (CI) were calculated. Descriptive statistics were used to summarize safety, pharmacokinetic, and pharmacodynamic outcomes. Statistical analyses were performed using SAS, version 9.4 (SAS Institute).

### Data Availability Statement

Upon request, and subject to review, Pfizer will provide the data that support the findings of this study. Subject to certain criteria, conditions, and exceptions, Pfizer may also provide access to the related individual de-identified participant data. See https://www.pfizer.com/science/clinical-trials/trial-data-and-results for more information.

## Results

### Preclinical Studies

**Figure 1:**
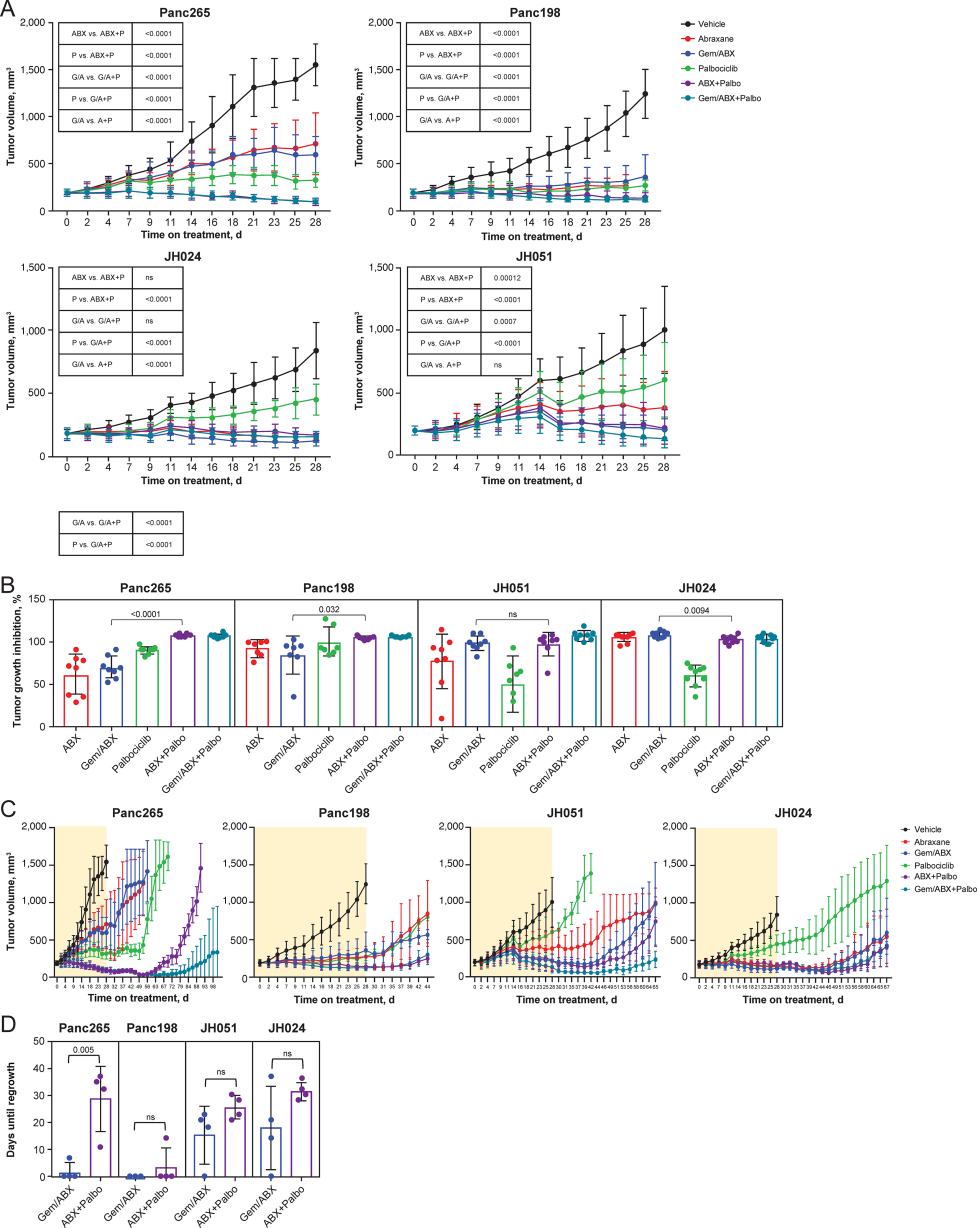
**A,** Tumor growth of the indicated PDAC PDX models treated with nab-paclitaxel (30 mg/kg; once weekly), gemcitabine (30 mg/kg once weekly)/nab-paclitaxel (30 mg/kg once weekly), palbociclib (100 mg/kg, 5 days/week) or the combinations following the scheme described in the Materials and Methods during 28 days. Data are mean ± SEM; *n* = 7–10 independent assays or replicas per group (ANOVA). **B,** TGI after 28 days of treatment in the indicated PDAC models treated as in **A**. Bars indicate mean ± SD (Student *t* test). **C,** Tumor growth of the indicated PDAC PDX models subjected to the indicated treatments. The yellow box indicates the period on treatment; mice were untreated from week 4 on. Data are mean ± SEM. **D,** Quantification of the delay until tumor regrowth, considered as time required for increase tumor size after end of treatment in the indicated PDAC PDX models. Horizontal bars represent mean ± SD (Student *t* test). ABX = abraxane; ANOVA = analysis of variance; G = gemcitabine; Gem = gemcitabine; ns = not significant; Palbo = palbociclib; PDAC = pancreatic ductal adenocarcinoma; PDX = patient-derived xenografts; SEM = standard error of the mean; TGI = tumor growth inhibition.

Palbociclib plus nab-paclitaxel was superior to GA in one of the four models tested, with a statistically higher tumor growth inhibition (Fig. 1). In addition, time to tumor progression was improved in the palbociclib-treated and nab-paclitaxel–treated groups, albeit findings were statistically significant in only one model. Palbociclib plus nab-paclitaxel was more effective against the Panc265 model. This is an aggressive, highly metastatic model with RAS and SMAD4 mutations that have been shown previously to be sensitive to the cell-cycle inhibitor dinaciclib (22–24). Notably, adding gemcitabine did not improve antitumor efficacy, supporting the evaluation of palbociclib plus nab-paclitaxel in a clinical trial.

### Patients and Treatment

In total, 76 patients were enrolled in the study (36 in the dose-escalation cohort, 20 in the modified dose–regimen cohorts, and 20 in the MTD cohort; Table 1). Among all patients, a median (range) of 4 (1‒21) palbociclib treatment cycles (28-day cycles, 3 weeks of treatment followed by 1 week off) was received. The median (range) duration of treatment was 3.7 (0‒20) months. The representativeness of the study population is in shown in Supplementary Table S1.

**TABLE 1:**
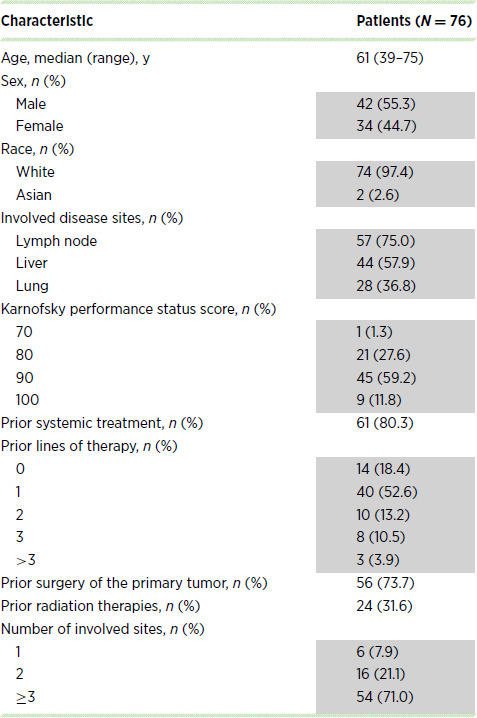
Patient demographics and baseline clinical characteristics

### Safety

A total of four dose-limiting toxicities were observed: 1 patient experienced grade 3 mucositis, 2 experienced grade 4 neutropenia, and 1 experienced febrile neutropenia. The MTD was palbociclib 100 mg for 21 of every 28 days and nab-paclitaxel 125 mg/m^2^ weekly for 3 weeks in a 28-day cycle. Among all patients, the most common all-causality any-grade AEs were neutropenia (76.3%), asthenia/fatigue (52.6%), nausea (42.1%), and anemia (40.8%; Table 2). All-causality grade 3/4 AEs were reported in 67 patients (88.2%); the most frequently reported grade 3/4 AEs were neutropenia (61.8%), leukopenia (26.3%), and anemia (22.4%; Table 2).

**TABLE 2:** All-causality AEs (≥10% of patients overall)

	Patients, *n* (%) (*N* = 76)	
Adverse event	Any grade	Grade 3/4
Any AE	76 (100.0)	67 (88.2)
Neutropenia	58 (76.3)	47 (61.8)
Asthenia/fatigue	40 (52.6)	9 (11.8)
Nausea	32 (42.1)	2 (2.6)
Anemia	31 (40.8)	17 (22.4)
Alopecia	30 (39.5)	0
Diarrhea	30 (39.5)	3 (3.9)
Leukopenia	25 (32.9)	20 (26.3)
Abdominal pain	24 (31.6)	3 (3.9)
Decrease appetite	24 (31.6)	1 (1.3)
Vomiting	23 (30.3)	3 (3.9)
Neurotoxicity/peripheral sensory neuropathy	19 (25.0)	7 (9.2)
Constipation	17 (22.4)	0
Pyrexia	17 (22.4)	0
Rash	16 (21.1)	0
Stomatitis	16 (21.1)	3 (3.9)
Back pain	13 (17.1)	1(1.3)
Arthralgia	10 (13.2)	0
Peripheral neuropathy	10 (13.2)	4 (5.3)
Thrombocytopenia	10 (13.2)	1 (1.3)
Dehydration	9 (11.8)	0
Dysgeusia	9 (11.8)	0
Cough	9 (11.8)	0
Disease progression	8 (10.5)	0
Headache	8 (10.5)	0
Lymphopenia	8 (10.5)	6 (7.9)

Abbreviation: AE, adverse event.

All-causality AEs associated with permanent and temporary discontinuation of palbociclib were reported in 13 (17.1%) and 63 (82.9%) patients, respectively, and AEs associated with palbociclib dose reduction were reported in 20 patients (26.3%). The AEs that led to permanent discontinuation of palbociclib were neoplastic progression (2.6%), gastrointestinal hemorrhage (2.6%), abdominal pain (1.3%), asthenia (1.3%), cardiac arrest (1.3%), cholangitis (1.3%), bacterial gastritis (1.3%), infection (1.3%), liver abscess (1.3%), peripheral neuropathy (1.3%), pain (1.3%), and sepsis (1.3%). Among these, 2 patients experienced AEs considered by the investigator to be treatment related: 1 patient experienced grade 3 peripheral neuropathy related to nab-paclitaxel, and 1 patient experienced grade 5 sepsis related to both palbociclib and nab-paclitaxel.

The most commonly reported treatment-related AEs among all patients were neutropenia (76.3%), alopecia (39.5%), and nausea (38.2%; Supplementary Table S2). Notably, treatment-related stomatitis (including aphthous stomatitis, cheilitis, glossitis, glossodynia, mouth ulceration, mucosal inflammation, oral pain, oropharyngeal discomfort, oropharyngeal pain, or stomatitis) was reported in 17.1% of patients. Diarrhea was experienced by 31.6% of patients, but most cases (91.7%) were mild or moderate. Fatigue was reported in 21.1% of patients (grade 1, 10.5%; grade 2, 9.2%; and grade 3, 1.3%). In the MTD cohort, neutropenia (90%), nausea (45%), and diarrhea (40%) were the most frequently reported treatment-related AEs (Supplementary Table S3).

### Pharmacokinetics

In the MTD cohort, the ratio of adjusted geometric mean (90% CI), as a percentage, for nab-paclitaxel administered alone and in combination with palbociclib was 107.88% (81.72‒142.42) for paclitaxel area under the plasma concentration–time curve from time 0 extrapolated to infinity (AUC_inf_) and 112.90% (68.32‒186.58) for the maximum observed concentration (*C*_max_). The ratios of adjusted geometric means (90% CIs) for nab-paclitaxel administered alone and in combination with palbociclib were 89.36% (76.62‒104.21) for paclitaxel AUC_inf_ and 87.98% (67.15‒115.25) for *C*_max_ in the overall population.

In the MTD cohort, the geometric mean *C*_max_ and area under the concentration–time profile from time 0 to time tau (τ), the dosing interval, where tau is 24 hours for once-daily dosing (AUCτ) at steady state, were 70.14 ng/mL and 1251 ng·hour/mL for palbociclib, respectively (Supplementary Table S4). The dose-normalized (to 125 mg) geometric mean *C*_max_ and AUCτ at steady state were 87.69 ng/mL and 1,564 ng·hour/mL, respectively. Results were similar in the overall population, with a dose-normalized geometric mean *C*_max_ of 90.44 ng/mL and AUCτ of 1,569 ng·hour/mL. These findings suggest no significant drug–drug interaction between palbociclib and nab-paclitaxel.

### Efficacy

Among the 23 patients receiving first-line treatment at the MTD who had not received prior chemotherapy in the metastatic setting (16 patients from the MTD cohort evaluable for antitumor activity and 7 first-line patients from the dose-escalation phase who received palbociclib 100 mg/nab-paclitaxel 125 mg/m^2^), the ORR was 13.0% and the clinical benefit rate was 65.2% (Table 3; Supplementary Fig. S2). Among all 27 patients receiving first-line treatment at the MTD, the median (95% CI) PFS was 5.3 (3.5–9.7) months, OS was 12.1 (6.4–14.8) months, and survival probability at 12 months was 50% (29.9–67.2). Among all patients, including those receiving doses other than the MTD, the median (95% CI) PFS was 3.8 (3.2–5.6) months, OS was 7.7 (6.3–10.3) months, and the survival probability at 12 months was 34.7% (24.2–45.4).

**TABLE 3:**
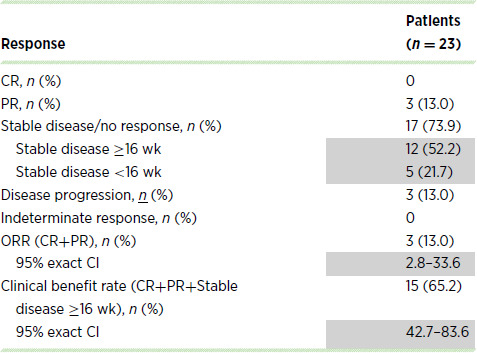
Antitumor activity among patients treated at the MTD

Abbreviations: CR, complete response; MTD, maximum tolerated dose; ORR, objective response rate; PR, partial response; wk, weeks.

### Pharmacodynamics and Biomarker Assessments

**Figure 2:**
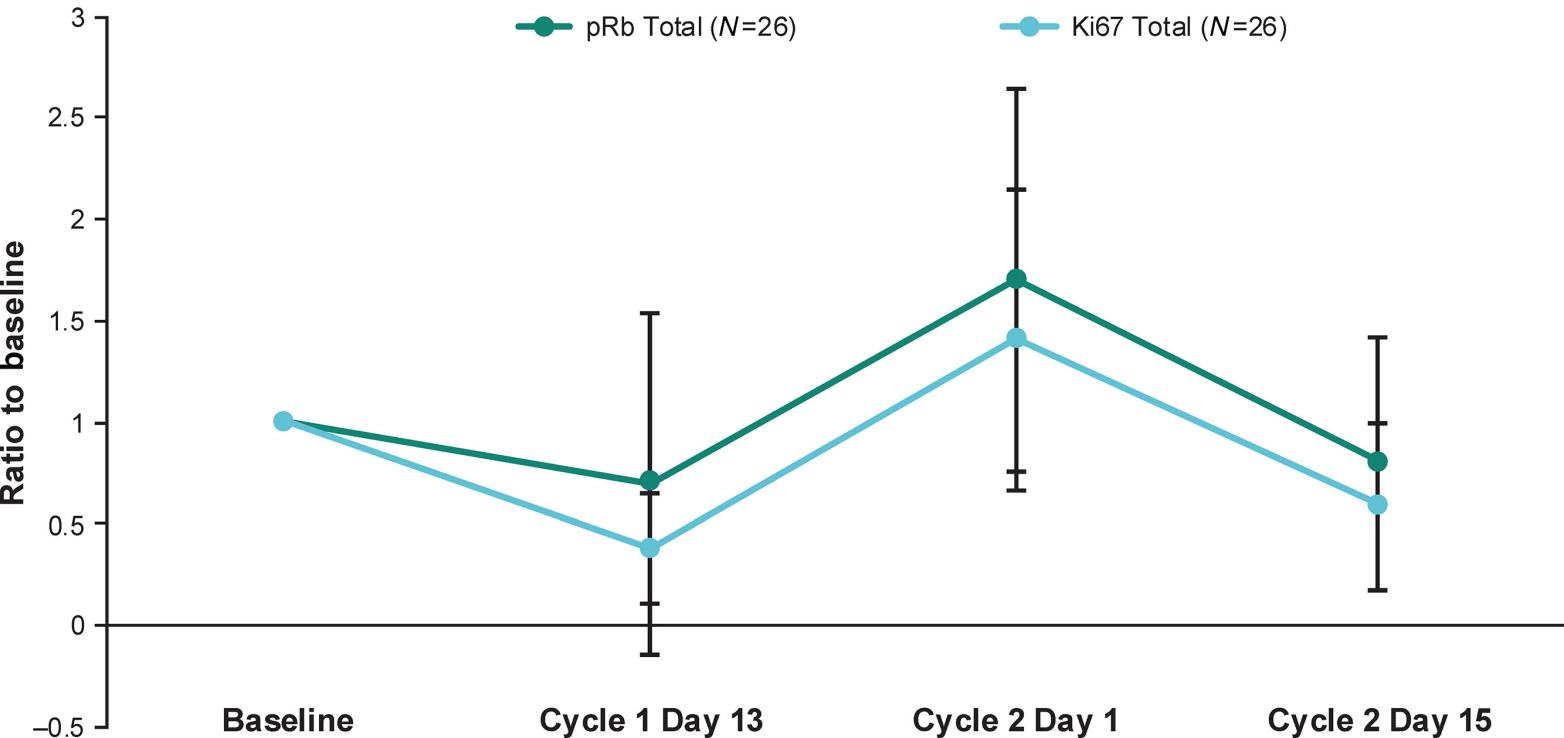
Ratio of pRb and Ki67 to baseline at three timepoints in serial paired skin biopsies. pRb = phosphor-Rb.

Of the 21 patients evaluable at the MTD, 11 (52.4%) had an on-treatment maximum Ca 19-9 reduction of >50% from baseline. The mean and median maximum Ca 19-9 reductions from baseline were 4924.3 and 402.6 U/mL, respectively. The pharmacodynamic activity of palbociclib plus nab-paclitaxel was shown by pRb and Ki67 modulation in serial paired skin biopsy specimens for 26 of the 27 patients in the first-line MTD treatment group (Fig. 2). pRb and Ki67 levels decreased after 2 weeks of palbociclib treatment and then rebounded to baseline or higher levels after the 1-week palbociclib break and before cycle 2 began. After a further 2 weeks of palbociclib treatment, levels decreased again to below baseline. Analysis of archived tumor samples using validated IHC assays, confirmed that both Rb and cyclin D1 were expressed in all patients in the MTD cohort.

Finally, molecular profiling based on circulating cfDNA using a validated next-generation sequencing assay revealed that the clinical outcome of patients without detectable *CDKN2A*, *RAS,* and *TP53* mutations (*n* = 58, 26, and 28 patients, respectively) was improved compared with patients with alterations detected (*n* = 12, 44, and 42; Supplementary Table S5). Both median PFS and OS were prolonged in patients without *CDKN2A*, *RAS,* and *TP53* mutations detected compared with those with alterations detected (Supplementary Table S5).

## Discussion

In preclinical studies, palbociclib was evaluated in a variety of combinations to inform the development of an appropriate approach to clinical evaluation for the treatment of PDAC. One objective of these studies was to establish the potential to reduce the burden of requiring a triple drug combination (palbociclib+nab-paclitaxel+gemcitabine). In a subset of these tumor models, the combination of palbociclib with either gemcitabine or nab-paclitaxel was evaluated to determine the optimal combination partner for palbociclib. Results from these studies have shown that the combination with nab-paclitaxel produced the dominant combinatorial effect with palbociclib, exceeding the antitumor effects of a GA regimen in two of three models and showing equivalence in the third model. In contrast, the addition of palbociclib to gemcitabine has yielded little sign of additivity. Consequently, a phase Ib trial was conducted to evaluate dose escalation, safety, pharmacokinetics, pharmacodynamics, and preliminary efficacy for palbociclib plus nab-paclitaxel in patients with advanced PDAC.

The current study aimed to determine the safety and efficacy of palbociclib in patients with advanced PDAC. On the basis of findings from preclinical studies conducted in PDX models, palbociclib plus nab-paclitaxel was evaluated in a clinical trial. The MTD was 100 mg/day palbociclib on days 1 to 21 plus 125 mg/m^2^ nab-paclitaxel on days 1, 8, and 15 of each 28-day cycle. There were no pharmacokinetic interactions, and the pharmacodynamic effects were as expected. At the MTD, the ORR was 13%, the median (95% CI) PFS survival was 5.3 (3.5–9.7) months, OS was 12.1 (6.4–14.8) months and the survival probability at 12 months was 50% (29.9–67.2). In addition, palbociclib plus nab-paclitaxel was associated with longer PFS and OS in the absence of detectable *CDKN2A, TP53,* and/or *RAS* alterations in plasma cfDNA.

As expected, the most common AEs associated with palbociclib plus nab-paclitaxel were hematologic; the incidence of grade 3/4 neutropenia (61.8%) was higher than reported with FOLFIRINOX (45.7%; ref. 3) or GA (38%; ref. 21). Neutropenia, however, was short lived and recovered to baseline levels and was rarely complicated with fever. Other nonhematologic grade 3/4 AEs were less common than reported with standard-of-care regimens (3, 21) and included peripheral neuropathy (5.3%), neurotoxicity (3.9%), diarrhea (3.9%), vomiting (3.9%), nausea (2.6%), and fatigue (2.6%). Interestingly, the occurrence of peripheral neuropathy was lower than reported previously with GA (17%; ref. 21), suggesting that this regimen could be useful in patients with preexisting nerve damage.

Paclitaxel is metabolized primarily by cytochrome P450 (CYP) 2C8 and CYP3A4 (25), and palbociclib is only a weak inhibitor of CYP3A4 (16). As expected, no significant pharmacokinetic interactions between palbociclib and nab-paclitaxel were identified. In the current study, the dose-normalized (to 125 mg) geometric mean of 90.44 ng/mL and AUC_τ_ of 1,569 ng·hour/mL at steady state were similar to values reported from the PALOMA-1 and PALOMA-2 trials of palbociclib in patients with breast cancer (AUC_τ_, 1,933 and 1,992 ng·hour/mL, respectively; *C*_max_, 108 and 110 ng/mL; ref. 26). The ratios of adjusted geometric means (90% CIs) for nab-paclitaxel administered alone and in combination with palbociclib were 89.36% (76.62‒104.21) for paclitaxel AUC_inf_ and 87.98% (67.15‒115.25) for *C*_max_. Pharmacodynamic analysis, using serial paired cells from skin biopsies as a proxy, showed that pRb and Ki67 levels decreased after 2 weeks of palbociclib treatment and then rebounded to baseline or higher levels after the 1-week palbociclib break. After a further 2 weeks of palbociclib treatment, levels again decreased to below baseline, observations consistent with CDK4/6 inhibition. Whether the same pharmacodynamic effects occur in tumor tissues and, if so, what the implications would be are unknown. Protracted target inhibition may be more effective, but because of toxicity issues, palbociclib cannot be administered continuously. Alternatively, and as discussed below, releasing cancer cells from cell-cycle arrest may increase vulnerability to treatment interventions.

Comparisons between efficacy results from the current study of palbociclib plus nab-paclitaxel and previous studies in patients with metastatic pancreatic cancer should be interpreted with caution but help to place our findings into perspective. The demographics of patients included in the previous open-label phase I/II study of GA and phase III study of GA compared with gemcitabine alone (21, 27) were generally similar to those in this study. In the phase III trial, GA resulted in an ORR of 23%, median PFS and OS of 5.5 and 8.5 months, respectively, and a 12-month survival rate of 35%. In the phase I/II trial of GA in patients with PDAC, the ORR was 48%, the median PFS was 7.9 months, the median OS was 12.2 months, and the 12-month survival rate was 48% (27). On the basis of data from the phase I/II trial, we set the prespecified efficacy target of ≥65% for 12-month survival rate. In the current study, the ORR was 13%, the median PFS was 5.3 months, the median OS was 12.1 months, and the survival probability at 12 months was 50% (21). Although the OS results in our current trial appeared encouraging, the low ORR and PFS suggest that the survival gain may be related to second-line treatment. Often, findings from early phase I and II trials with novel regimens in PDAC conducted at academic centers are interpreted by comparing results with worldwide phase III trials and, not surprisingly, the comparison appears favorable, leading to phase III results that are then negative. For the current study, we used early phase II data and set a higher target for phase III development. This strategy may help prioritize developing regimens with real promise to be effective.

Despite the strong rationale based upon *CDKN2A* common alterations in PDAC and strong preclinical data, the CDK inhibitor palbociclib did not have meaningful clinical activity in *CDKN2A-*mutated advanced pancreatic cancer in a previous study (28), and palbociclib plus nab-paclitaxel did not reach the expected level of efficacy in our study. To better understand the mechanism underlying these findings, additional extensive preclinical studies in PDX, organoids, and genetically engineered mouse modes of PDAC have been conducted (29). These studies revealed a clear sequence-dependent interaction between a variety of chemotherapy agents often used in pancreatic cancer and CDK4/6 blockade (29). Inhibition of CDK4/6 after chemotherapy impaired homologous recombination, leading to DNA damage (29). Furthermore, this approach may sensitize tumors to PARP inhibitors as well (29). Notably, concomitant administration of CDK inhibitors and chemotherapy was less effective and, in some models, even provided protection from chemotherapy-induced cell death (29). Indeed, chemotherapy-induced resistance via *CDK4* amplification, *RB* loss, and cyclin E1 amplification has been suggested to contribute to the lack of efficacy of palbociclib in pancreatic cancer when given as the third or fourth line of treatment (28). Future clinical trials are planned to evaluate the optimal sequence of treatments.

## Conclusions

This phase Ib study demonstrated the tolerability of palbociclib plus nab-paclitaxel for patients with PDAC. Antitumor activity was observed with the combination regimen but did not meet the prespecified efficacy threshold. Although some patients benefited from treatment, the lack of a validated biomarker for patient selection limits the clinical usefulness of these data. Additional preclinical studies suggest a sequence-dependent interaction between chemotherapy and CDK4/6 blockade that should be explored in future clinical trials.

## Supplementary Material

Supplementary Materials and Methods Supplementary Materials and Methods

Supplementary Figure S1 A) Palbociclib and nab-paclitaxel combination dose escalation and de-escalation sequence. B) Number of patients with dose-limiting toxicities for dose escalation decisions at a dose level.

Supplementary Figure S2 Swimmer's Plot of Best Response.

Supplementary Table S1 Supplementary Table S1.pdf

Supplementary Table S2 Treatment-Related Any-Grade AEs (≥10% of Patients Overall).

Supplementary Table S3 Supplementary Table S3.pdf

Supplementary Table S4 Palbociclib Pharmacokinetic Parameters in the MTD Cohort.

Supplementary Table S5 Summary of PFS and OS by Mutation Status.
