# Supplementary material for: A Preclinical and Phase Ib Study of Palbociclib plus Nab-Paclitaxel in Patients with Metastatic Adenocarcinoma of the Pancreas
Source: Cancer Res Commun. 2022 Nov 2;2(11):1326–33. doi: 10.1158/2767-9764.CRC-22-0072 (PMC10035387; doi:10.1158/2767-9764.CRC-22-0072)
Supplement: Supplementary Materials and Methods [file crc-22-0072-s01.pdf]

Online-Only Supplemental Materials

Supplementary Materials and Methods

Supplementary Table S1. Representativeness of Study Population

Supplementary Table S2. Treatment-Related Any-Grade AEs (≥10% of Patients Overall)

Supplementary Table S3. Treatment-Related Any-Grade AEs Among Patients in the MTD Cohort (≥10% of Patients)

Supplementary Table S4. Palbociclib Pharmacokinetic Parameters Among Patients in the MTD Cohort

Supplementary Table S5. Summary of PFS and OS by Mutation Status

Supplementary Figure S1. A) Palbociclib and Nab-Paclitaxel Combination Dose Escalation and De-escalation Sequence, **B)** Number of Patients with DLT for Dose Escalation Decisions at a Dose Level

DLT=dose-limiting toxicity.

In the figure, the term “DLT” means “patients with DLT,” not the actual number of DLT events.

Supplementary Figure S2. Swimmer’s Plot of Best Response

Supplementary Materials and Methods

Preclinical Data

Cohort establishment

Female athymic nude mice (aged 6–8 weeks; 15–20 g) purchased from Charles River Laboratories (Wilmington, MA, USA) were used for patient-derived xenografts (PDX) modeling. Frozen PDX tissue was thawed and then cut into 4 × 4 × 4-mm pieces. Tumor pieces were incubated in Matrigel (BD) for 10 minutes, and each piece was subcutaneously inserted into the flank of each mouse. After 2–3 months, tumors were approximately 500 mm^3 in volume. At this moment, tumors were expanded using the same protocol used above, but with smaller tumor pieces (2 × 2 × 2 mm).

Tumor measurement

Tumor volume was determined using a caliper measuring the shorter and larger diameters. Considering tumors as an ellipse, we applied the following formula to estimate the volume: (short x short x large)/2. When subcutaneous tumors reached 150-250 mm^3 in volume mice, they were randomized in the different groups of treatment, including at least 6 tumors per group. Tumors were measured 3 times weekly.

Mouse treatment

Mice were treated with palbociclib (provided by Pfizer Inc, New York, NY, USA) 100 mg/kg resuspended in lactate buffer pH4 oral gavage 5 days on, 2 days off; nab-paclitaxel (Accord, Durham, NC, USA) 30 mg/kg in physiologic serum intravenously once weekly; and gemcitabine (Lilly USA, Indianapolis, IN, USA) 30 mg/kg in physiologic serum intraperitoneally in

combination with nab-paclitaxel once weekly for combination gemcitabine/nab-paclitaxel or nab-paclitaxel administered 1 day; palbociclib was administered the next 5 days and 1 holiday. That cycle was repeated during the 28 days of experiment.

Biomarker Analysis

Voluntary skin biopsy specimens for PD assessments were collected predose on Day –2 of cycle 1, 6 hours post-palbociclib dose on Day 13 of cycle 1, and pre-palbociclib and nab-paclitaxel doses on Day 1 of cycle 2 and Day 15 of cycle 2. For biopsy specimens taken at Day 13 of cycle 1 and Day 15 of cycle 2, patients must have received ≥7 consecutive days of palbociclib.

For pRb, a rabbit monoclonal primary antibody, Phospho-Rb (Ser807/811) (D20B12) XP Rabbit mAb (Cell Signaling #8516, Danvers, MA, USA), was used to perform the staining on the Ventana Benchmark Ultra (Q^2 Solutions, Valencia, CA, USA). The assay specifically detected the pRb protein localized in the nuclei of cells that expressed the pRb antigen. IHC scoring was based on intensity of staining and percentage of stained targeted squamous epithelium cells of corresponding staining intensity, quantified using H-scores. For Ki67, the Ventana CONFIRM™ anti-Ki-67 (30-9) rabbit monoclonal primary antibody was used to perform staining on the Ventana Benchmark Ultra; the percentage of Ki-67–positive cells was calculated based on the number of Ki-67–positive cells and the number of cells counted, intensity of staining was not taken into consideration. The proliferation index cutoff used was 30%, with ≥30% corresponding to high proliferation index and >30% corresponding to low proliferation index.

Exploratory Analyses

Blood specimens optimized for plasma preparation for nucleic acid analysis were collected Day –2 of cycle 1 (predose), Day 1 of cycle 3 (before the first administration of investigational treatments, aligned with the first tumor assessment on study treatment), and at the end of treatment.
