# Supplementary material for: A Preclinical and Phase Ib Study of Palbociclib plus Nab-Paclitaxel in Patients with Metastatic Adenocarcinoma of the Pancreas
Source: Cancer Res Commun. 2022 Nov 2;2(11):1326–33. doi: 10.1158/2767-9764.CRC-22-0072 (PMC10035387; doi:10.1158/2767-9764.CRC-22-0072)
Supplement: Supplementary Figure S1 — A) Palbociclib and nab-paclitaxel combination dose escalation and de-escalation sequence. B) Number of patients with dose-limiting toxicities for dose escalation decisions at a dose level. [file crc-22-0072-s02.pdf]

Supplementary Figure S1.

A) Palbociclib and nab-paclitaxel combination dose escalation and de-escalation sequence

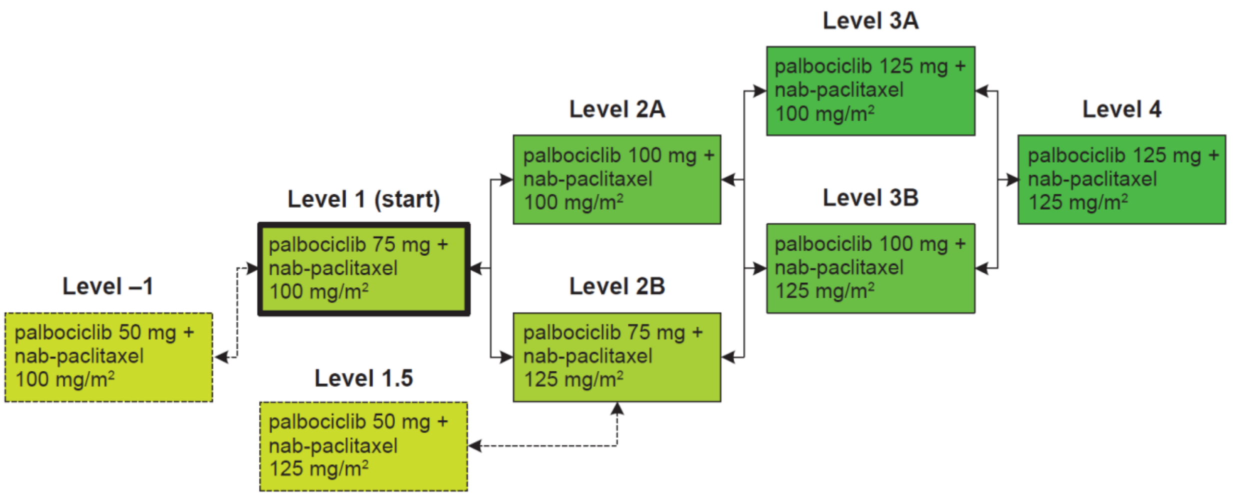

B) Number of patients with dose-limiting toxicities for dose escalation decisions at a dose level

	**Total Number of DLT Evaluable Patients**								
	**2**	**3**	**4**	**5**	**6**	**7**	**8**	**9**	**10**
**Escalate**	0	0	0	0	0–1	0–1	0–1	0–2	0–2
**Stay**	1	1	1	1–2	2	2	2		3
**De-Escalate & revisit allowed**		2	2			3	3–4	3–4	4–5
**De-Escalate & revisit not allowed**	2	3	3–4	3–4	3–5	4–5	5	5	6

DLT=dose-limiting toxicity.

In the figure, the term “DLT” means “patients with DLT,” not the actual number of DLT events.
