# Supplementary figures and images for: A Preclinical and Phase Ib Study of Palbociclib plus Nab-Paclitaxel in Patients with Metastatic Adenocarcinoma of the Pancreas
Source: Cancer Res Commun. 2022 Nov 2;2(11):1326–33. doi: 10.1158/2767-9764.CRC-22-0072 (PMC10035387; doi:10.1158/2767-9764.CRC-22-0072)

Supplementary Figure S2. Swimmer's Plot of Best Response

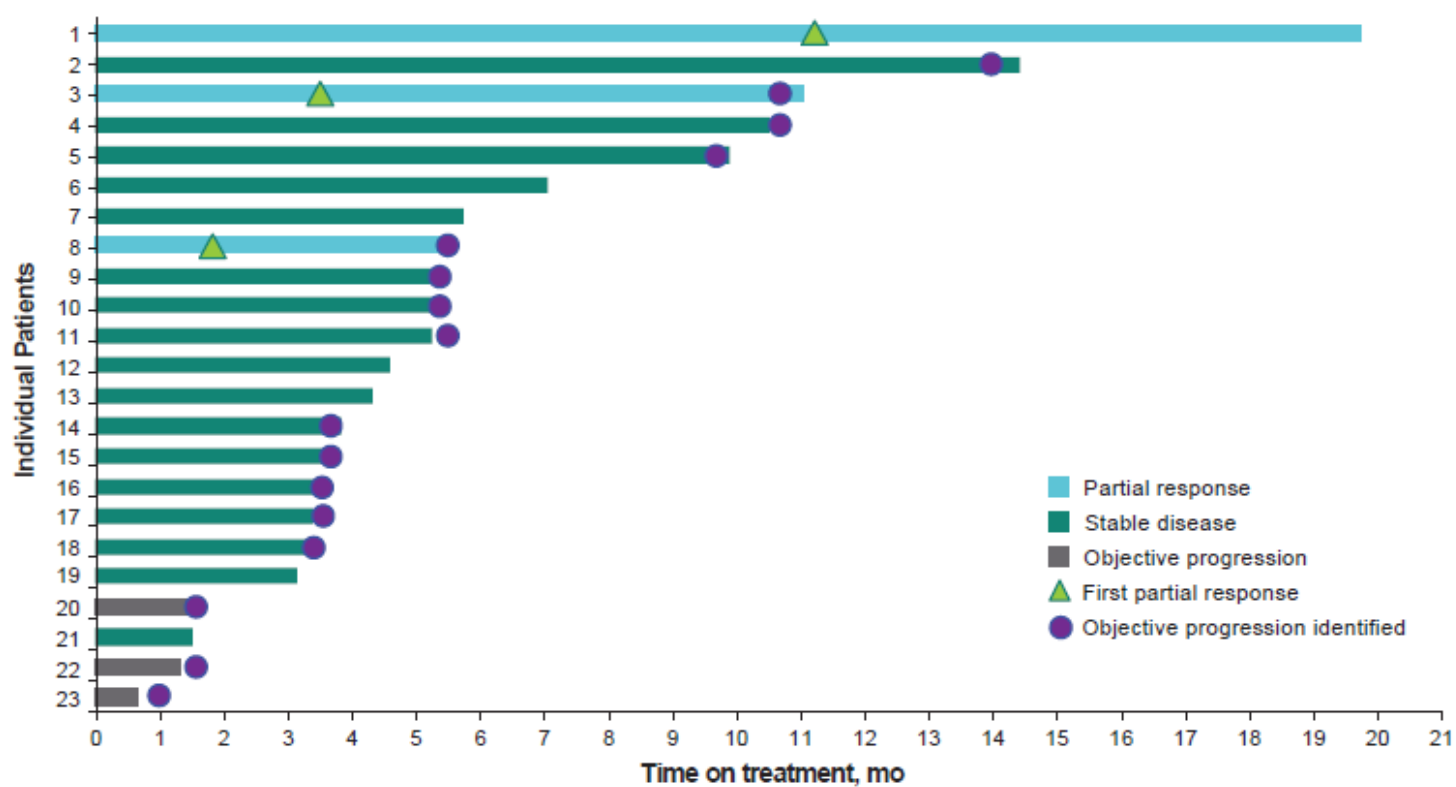

Supplement: Supplementary Figure S2 — Swimmer's Plot of Best Response. [file crc-22-0072-s03.pdf]
