# Supplementary material for: A Preclinical and Phase Ib Study of Palbociclib plus Nab-Paclitaxel in Patients with Metastatic Adenocarcinoma of the Pancreas
Source: Cancer Res Commun. 2022 Nov 2;2(11):1326–33. doi: 10.1158/2767-9764.CRC-22-0072 (PMC10035387; doi:10.1158/2767-9764.CRC-22-0072)
Supplement: Supplementary Table S1 [file crc-22-0072-s04.pdf]

Supplementary Table S1. Representativeness of Study Participants

**Considerations related to:**	**Pancreatic Ductal Adenocarcinoma (PDAC)**
Sex	The incidence of PDAC is higher in males than females, 9.5/1,000 and 7.0/1,000, respectively. Male gender has been shown to be a risk factor for PDAC.
Age	The most frequent age of diagnosis for both genders is 60–69 years.
Race/ethnicity	Considerably higher incidence of PDAC in Black patients have been reported in studies from the United States and this is reflected by mortality rates. Black race has been found to be a risk factor for PDAC.
Geography	Europe has the highest age-standardized PDAC mortality rate, followed by North America. Africa and Central America have the lowest PDAC mortality rates.
Other considerations	The major risk factors for PDAC are family history, genetic disorders, complications and preferences.
Overall representativeness of this study	The age distribution and gender of our study population, median age 61 years and 55% male, reflect the demographics of PDAC in the general population. Our study population was relatively small (N=76) and patient recruitment was limited to the centers participating in the study, and thus no Black patients were recruited.
