# Supplementary material for: A Preclinical and Phase Ib Study of Palbociclib plus Nab-Paclitaxel in Patients with Metastatic Adenocarcinoma of the Pancreas
Source: Cancer Res Commun. 2022 Nov 2;2(11):1326–33. doi: 10.1158/2767-9764.CRC-22-0072 (PMC10035387; doi:10.1158/2767-9764.CRC-22-0072)
Supplement: Supplementary Table S2 — Treatment-Related Any-Grade AEs (≥10% of Patients Overall). [file crc-22-0072-s05.pdf]

Supplementary Table S2. Treatment-Related Any-Grade AEs (≥10% of Patients Overall)

	Patients (N=76)
Any AEs	75 (98.7)
Neutropenia	58 (76.3)
Alopecia	30 (39.5)
Nausea	29 (38.2)
Diarrhea	24 (31.6)
Leukopenia	23 (30.3)
Anemia	21 (27.6)
Decreased appetite	17 (22.4)
Asthenia	16 (21.1)
Fatigue	16 (21.1)
Vomiting	15 (19.7)
Rash	14 (18.4)
Stomatitis	13 (17.1)
Neurotoxicity	11 (14.5)
Peripheral neuropathy	10 (13.2)
Thrombocytopenia	9 (11.8)
Dysgeusia	8 (10.5)
Peripheral sensory neuropathy	8 (10.5)

AE=adverse event.
