# Supplementary material for: A Preclinical and Phase Ib Study of Palbociclib plus Nab-Paclitaxel in Patients with Metastatic Adenocarcinoma of the Pancreas
Source: Cancer Res Commun. 2022 Nov 2;2(11):1326–33. doi: 10.1158/2767-9764.CRC-22-0072 (PMC10035387; doi:10.1158/2767-9764.CRC-22-0072)
Supplement: Supplementary Table S3 [file crc-22-0072-s06.pdf]

Supplementary Table S3. Treatment-Related Any-Grade AEs Among Patients in the MTD Cohort (≥10% of Patients)

	**Patients (n=20)**
Any AEs	20 (100.0)
Neutropenia	18 (90.0)
Nausea	9 (45.0)
Diarrhea	8 (40.0)
Alopecia	6 (30.0)
Leukopenia	6 (30.0)
Rash	6 (30.0)
Anemia	5 (25.0)
Fatigue	5 (25.0)
Thrombocytopenia	5 (25.0)
Dry skin	4 (20.0)
Peripheral neuropathy	4 (20.0)
Neurotoxicity	4 (20.0)
Asthenia	3 (15.0)
Constipation	3 (15.0)
Lymphopenia	3 (15.0)
Vomiting	3 (15.0)
Decreased appetite	2 (10.0)
Dehydration	2 (10.0)
Headache	2 (10.0)
Peripheral sensory neuropathy	2 (10.0)
Stomatitis	2 (10.0)

AE=adverse event; MTD=maximum-tolerated dose.
