# Supplementary material for: A Preclinical and Phase Ib Study of Palbociclib plus Nab-Paclitaxel in Patients with Metastatic Adenocarcinoma of the Pancreas
Source: Cancer Res Commun. 2022 Nov 2;2(11):1326–33. doi: 10.1158/2767-9764.CRC-22-0072 (PMC10035387; doi:10.1158/2767-9764.CRC-22-0072)
Supplement: Supplementary Table S4 — Palbociclib Pharmacokinetic Parameters in the MTD Cohort. [file crc-22-0072-s07.pdf]

Supplementary Table S4. Palbociclib Pharmacokinetic Parameters in the MTD Cohort

	Patients (n=15)		
	**Geometric Mean**	**Geometric CV, %**	**Median**
AUC_{τ}, ng▪hr/mL	1251	38	1350
CL/F, L/h	79.95	38	74.10
C_{max}, ng/mL	70.14	43	69.10
C_{trough}, ng/mL	34.89	34	34.60
t_{max}, hr	NA	NA	4.17

AUC_{τ}=area under the concentration-time profile from time 0 to time τ, the dosing interval, where τ=24 hours; CL/F=apparent oral clearance; C_{max}=maximum plasma concentration at steady state; C_{trough}=predose concentration; MTD=maximum-tolerated dose; NA=not available; t_{max}=time for C_{max}.
