# Supplementary material for: A Preclinical and Phase Ib Study of Palbociclib plus Nab-Paclitaxel in Patients with Metastatic Adenocarcinoma of the Pancreas
Source: Cancer Res Commun. 2022 Nov 2;2(11):1326–33. doi: 10.1158/2767-9764.CRC-22-0072 (PMC10035387; doi:10.1158/2767-9764.CRC-22-0072)
Supplement: Supplementary Table S5 — Summary of PFS and OS by Mutation Status. [file crc-22-0072-s08.pdf]

**Supplementary Table S5. Summary of PFS and OS by Mutation Status**

| | Median PFS (95% CI), mo | HR[a] (95% CI) | Median OS (95% CI), mo | HR[a] (95% CI) |
|---|---|---|---|---|
| *CDKN2A* mutation[b] | | | | |
| Detected (n=12) | 1.9 (1.6–5.3) | 0.439 (0.221–0.874) | 5.1 (2.4–7.2) | 0.328 (0.169–0.635) |
| Not detected (n=58) | 5.5 (3.5–6.7) | | 9.8 (6.9–13.0) | |
| *RAS* mutation[b] | | | | |
| Detected (n=44) | 3.5 (1.9–5.3) | 0.428 (0.239–0.767) | 6.3 (5.3–7.4) | 0.313 (0.174–0.562) |
| Not detected (n=26) | 8.6 (3.8–10.9) | | 15.5 (10.3–22.3) | |
| *TP53* mutation[b] | | | | |
| Detected (n=42) | 3.6 (1.9–5.3) | 0.420 (0.237–0.744) | 6.3 (5.4–8.8) | 0.316 (0.173–0.577) |
| Not detected (n=28) | 9.1 (3.4–10.9) | | 15.1 (7.4–22.3) | |

HR=hazard ratio; OS=overall survival; PFS=progression-free survival.

[a]HR based on Cox proportional hazards model; under the proportional hazards assumption, a HR <1 indicates a lower risk of disease progression or death in the group of patients in whom the indicated biomarker mutation was not detected.

[b]Detected in peripheral blood specimens using plasma circulating nuclei acid mutational
